# *Mycoplasma bovis* Invades Non-Phagocytic Cells by Clathrin-Dependent Endocytic Pathways and Escapes from Phagocytic Vesicles

**DOI:** 10.3390/pathogens13111003

**Published:** 2024-11-15

**Authors:** Bin Li, Yabin Lu, Yaru Feng, Xiaolong Jiao, Qiuyu Zhang, Mengting Zhou, Yuyu Zhang, Jian Xu, Yuefeng Chu, Duoliang Ran

**Affiliations:** 1 College of Veterinary Medicine, Xinjiang Agricultural University, Urumqi 830052, China; libin19921221@126.com (B.L.); lyb4095@163.com (Y.L.); fengyaru1998@163.com (Y.F.); 18299128126@163.com (X.J.); zqy1141665974@163.com (Q.Z.); 18299383302@163.com (M.Z.); zhangyuyu2000xj@163.com (Y.Z.); xujian@caas.cn (J.X.); 2 Xinjiang Key Laboratory of New Drug Research and Development for Herbivores, Urumqi 830052, China; 3 State Key Laboratory for Animal Disease Control and Prevention, College of Veterinary Medicine, Lanzhou University, Lanzhou Veterinary Research Institute, Chinese Academy of Agricultural Sciences, Lanzhou 730000, China

**Keywords:** *Mycoplasma bovis*, invasion, endocytic, immune escape, clathrin

## Abstract

*Mycoplasma bovis* (*M. bovis*) is capable of causing pneumonia, arthritis, mastitis, and various other ailments in cattle of all age groups, posing a significant threat to the healthy progression of the worldwide cattle industry. The invasion of non-phagocytic host cells serves as a pivotal mechanism enabling *M. bovis* to evade the immune system and penetrate mucosal barriers, thereby promoting its spread. To investigate the differences in *M. bovis* invasion into four types of non-phagocytic cells (Madin–Darby bovine kidney (MDBK) cells, embryonic bovine lung (EBL) cells, bovine embryo tracheal (EBTr) cells and bovine turbinate (BT) cells) and further elucidate its invasion mechanism, this study first optimized the experimental methods for *M. bovis* invasion into cells. Utilizing laser scanning confocal microscopy, transmission electron microscopy, and high-content live-cell imaging systems, the invasion process of *M. bovis* into four types of non-phagocytic cells was observed. The invasion rates of three different strains of *M. bovis* (PG45, 07801, 08M) were quantified through the plate counting method. In order to clarify the specific pathway of *M. bovis* invasion into cells, chlorpromazine (CPZ), amiloride (AMI), and methyl-β-cyclodextrin (M-β-CD) were used to inhibit CLR-mediated clathrin-dependent endocytosis (CDE) pathway, macropinocytosis, and lipid raft pathway, respectively. Subsequently, the invasion rates of PG45 into these four types of cells were measured. Using siRNA technology, the expression of clathrin (CLR) in EBL cells was knocked down to further verify the role of CLR in the invasion process of *M. bovis*. The results showed that the optimal conditions for *M. bovis* to invade non-phagocytic cells were a multiplicity of infection (MOI) of 1000 and an optimal invasion time of 4 h. All three strains of *M. bovis* have the ability to invade the four types of non-phagocytic cells, yet their invasion abilities vary significantly. Observations from transmission electron microscopy further confirmed that at 120 min post-infection, PG45 had successfully invaded EBL cells and was present within endocytic vesicles. It is noteworthy that almost all PG45 successfully escaped from the endocytic vesicles after 240 min of infection had passed. Through chemical inhibition experiments and CLR protein knockdown experiments, it was found that when the CDE and lipid raft pathways were blocked or CLR protein expression was reduced, the invasion rates of PG45, 07801, and 08M in MDBK, EBL, EBTr, and BT cells were significantly decreased (*p* < 0.05). The above results indicate that *M. bovis* can invade all types of non-phagocytic cells through endocytic pathways involving CDE (clathrin-dependent endocytosis) or lipid raft-mediated endocytosis, and possesses the ability to escape from phagosomes.

## 1. Introduction

*Mycoplasma bovis* is the smallest organism that reproduces independently; it is approximately 200–400 nm in size and has no cell wall. Because it cannot synthesize some amino acids, nucleic acid precursors and cholesterol [1], its requirements for nutrients in the culture medium are relatively high. *M. bovis* can infect a variety of bovine species of almost all ages, including cows, yellow cattle, buffaloes, yaks, pronghorns, and other ruminants [2,3,4]. *M. bovis* infection often causes bovine pneumonia [5], mastitis [6], arthritis [7], keratitis [8], otitis media [9] and abortion [10]. The pathogen was first isolated from cows with American dairy cow mastitis in 1961 [11]. It has now developed into a worldwide epidemic and has brought huge economic losses to the global cattle industry. According to statistics, the economic losses due to calf pneumonia caused by *M. bovis* infection amount to approximately EUR 1.04–1.97 million per year in Europe and USD 140 million per year in the Americas [12,13].

The invasion of all types of host cells is an effective way to establish continuous infection and immune evasion [14,15]. In particular, the invasion of all types of non-phagocytes (especially epithelial cells) is a common strategy for most chronically infectious pathogens [16]. Invading host cells can not only help pathogens to quickly pass through the organism’s immune barrier but also allows them to effectively avoid direct contact with immune cells and achieve immune evasion [17]. Pathogenic bacteria can express various surface proteins and bind to host receptors [18,19,20]. The signal is often transduced by triggering the ubiquitination pathway through the interaction between the surface proteins and the host receptors [21]. Actin recruitment by regulatory and host factors leads to cytoskeletal rearrangement. Subsequently, bacteria enter host cells by mediating internalization [22]. *Streptococcus agalactiae*, *Staphylococcus aureus* [23] and *Mycoplasma pneumoniae* [24] achieve their invasion by binding to the extracellular matrix (ECM). For example, *M. bovis* combines with calcium-dependent phospholipid-binding protein annexin 2 to adhere and invade embryonic bovine lung (EBL) cells [25].

Although *M. bovis* encodes fewer proteins and lacks the toxins and two-component system that are widely found in bacteria, it can damage host cells and evade the host’s immune system through multiple pathways, allowing it to pass through the immune barrier into the host and trigger various types of inflammation [26]. Numerous research studies have demonstrated that mycoplasmas are able to invade or internalize a variety of non-phagocytes [17], such as *M. penetrants*, *M. fermentans*, *M. pneumoniae* and *M. bovis*, among others. It either enters host cells or localizes throughout the cytoplasm and perinuclear regions [27]. However, its invasion mechanism still needs further analysis.

*M. bovis* can avoid direct contact with immune molecules and drugs by invading various non-phagocytic cells and surviving within them. However, the current research on the pathways of *M. bovis* invasion into non-phagocytic cells and its survival within these cells is still not thorough. Therefore, this study aims to observe the differences in the invasion abilities of various *M. bovis* strains into four types of non-phagocytic cells, and simultaneously understand the potential important roles of clathrin-mediated endocytosis and lipid raft pathways in the invasion process of *M. bovis*. This provides a new scientific basis for revealing the pathways and mechanisms of *M. bovis* survival within cells.

## 2. Materials and Methods

### 2.1. Cells and Bacterial Strain

Four different epithelial cell lines were used: Embryonic Bovine Lung cells (EBL) (CVCL_2028) [kindly donated by Pro. Xiaochun Wu (Gansu Agricultural University, Lanzhou, Gansu, China)], Bovine Embryo Tracheal cells (EBTr) (CVCL_2442) and Bovine Turbinate cells (BT) (CVCL_4134) [kindly donated by Pro. Xingwen Bai (Lanzhou Veterinary Research Institute, Lanzhou, Gansu, China)], and Madin-Darby Bovine Kidney cells (MDBK) (CVCL_0421) were obtained from the Cell Resource Center, Peking Union Medical College (PCRC), and preserved in the laboratory. All cells were cultured in Dulbecco’s Modified Eagle Medium (DMEM) (Thermo Fisher Scientific, Waltham, MA, USA) with L-glutamine and Phenol Red supplemented with 10% fetal bovine serum (FBS) (Thermo Fisher Scientific, Waltham, MA, USA). Cells were maintained at 37 °C with 5% CO_2_. In all cases, the cell medium used was low-glucose.

*M. bovis* PG45 (ATCC 25523) was preserved in the laboratory. *M. bovis* 08M (GenBank: CP019639.1) was provided by the State Key Laboratory of Veterinary Etiological Biology, Lanzhou Veterinary Research Institute, Chinese Academy of Agricultural Sciences. *M. bovis* 08701 was isolated from bovine clinical samples and stored in our laboratory. The PG45-GFP (Green fluorescent protein) strain, which was previously successfully constructed in our laboratory and is capable of stably expressing GFP fluorescein protein and stimulating green fluorescence, is stored by our laboratory (unpublished data). All the aforementioned *M. bovis* strains are stored at −80 °C. Prepare the Modified Thiaucourt’s Broth (MTB) and Modified Thiaucourt’s Agar (MTA) plate. The recipe for MTB medium is as follows: 18 g of PPLO Broth (Thermo Fisher Scientific), 1 g of glucose (Sigma-Aldrich, St. Louis, MO, USA), 1 g of sodium glutamate (Sigma-Aldrich, St. Louis, MO, USA), 100 mL of yeast extract (Thermo Fisher Scientific, Waltham, MA, USA), and 5 mL of 10% phenol red solution. The mixture should be autoclaved at 121 °C for 20 min. After cooling, add 100 mL of horse serum (Thermo Fisher Scientific, Waltham, MA, USA) and 200,000 units of penicillin (Thermo Fisher Scientific, Waltham, MA, USA) The formulation for MTA medium is as follows: 22 g of PPLO agar (Thermo Fisher Scientific, Waltham, MA, USA), 1 g of glucose (Sigma-Aldrich, St. Louis, MO, USA), 1 g of sodium glutamate (Sigma-Aldrich, St. Louis, MO, USA), and 100 mL of yeast extract (Thermo Fisher Scientific, Waltham, MA, USA). The mixture should be autoclaved at 121 °C for 20 min. After cooling, add 100 mL of horse serum (Thermo Fisher Scientific, Waltham, MA, USA) and 200,000 units of penicillin (Thermo Fisher Scientific, Waltham, MA, USA). *M. bovis* was grown in MTB medium at 37 °C with 5% CO_2_ for 48 h. The method for calculating colony-forming units (CFU) of *M. bovis* involves counting individual colonies under an optical microscope. Initially, 50 mL of *M. bovis* in the logarithmic growth phase is taken and centrifuged at 10,000× *g* for 15 min. After discarding the supernatant, the pellet is resuspended in 5 mL of MTB. Then, 100 μL of this suspension is subjected to serial dilution. The diluted samples are then spread onto MTA. The plates are incubated in a 5% CO_2_ incubator at 37 °C for 4 days. Subsequently, individual colonies are counted under an optical microscope.

### 2.2. Minimum Bactericidal Concentration

The minimum bactericidal concentration (MBC) was determined using the agar method [28]. A broth culture of PG45 after 48 h of incubation was used as an inoculum for MBC determination. PG45 (2.5 × 10^8^ CFU) was treated with tetracycline (Med Chem Express, Monmouth Junction, NJ, USA), azithromycin (Med Chem Express), gentamicin (Med Chem Express) and tiamulin fumarate (Med Chem Express) solutions at 37 °C with 5% CO_2_ for 3 h. All antibiotics were diluted to a working concentration from 50 µg/mL to 1000 µg/mL (50, 100, 200, 400, 1000). In order to remove each antibiotic, we collected all media, centrifuged them for 15 min at 10,000× *g* and washed them three times with MTB. The culture medium was added to MTA plates at 37 °C with 5% CO_2_ for 4 days to calculate the MBC. The concentration of the antimicrobial agent without colony formation was defined as the MBC.

Using the same experimental protocol, 2.5 × 10^8^ CFU PG45 was treated with tetracycline, azithromycin, gentamicin and tiamulin fumarate for 2 h, 3 h and 4 h. The antibiotics were diluted to 200 µg/mL and 400 µg/mL. All experiments were repeated at least three times.

### 2.3. Cell Invasion Assay

The EBL cells were subcultured into 12-well cell plates and grown until to a confluence of 80–90% and infected with PG45 (MOI of 0.1, 1, 10, 50, 100, 500, 1000, 5000 and 10,000). After incubation at 37 °C with 5% CO_2_ for 4 h, the unadhered PG45 were washed off with pre-warmed 0.01 M phosphate-buffered saline (PBS) (dissolve 8 g of NaCl, 0.2 g of KCl, 1.44 g of Na_2_HPO_4_, and 0.24 g of KH_2_PO_4_ in 800 mL of distilled water, adjust the pH of the solution to 7.4 with HCl, and finally add distilled water to bring the volume to 1 L) and treated with 400 µg/mL of gentamicin at 37 °C for 4 h to kill the extracellular PG45. The cells were washed with PBS and digested using 0.25% trypsin–ethylenediaminetetraacetic acid (EDTA), and the invasive mycoplasmas were counted using MTA plates.

For the invasion time optimized assay, EBL cells were infected with PG45 (MOI of 1000) to determine the invasion rates at 15 min, 30 min, 60 min, 90 min, 120 min, 180 min, 240 min and 480 min. Each experiment was conducted a minimum of three times to ensure reproducibility.

### 2.4. Intracellular Survival Assay

EBL cells were cultured until subconfluent in a 6-well cell culture plate and were infected with 2.5 × 10^8^ CFU/well (MOI of 1000) of PG45 (our research confirmed that 400 µg/mL gentamicin solution could kill PG45 at a density of 2.5 × 10^8^ CFU/well). At 4 h after infection at 37 °C and 5% CO_2_, the EBL cells were washed three times with 0.01 M PBS per well to remove non-adherent PG45. To determine the number of intracellular PG45, the EBL cells were treated with 400 µg/mL gentamicin solution at 37 °C and 5% CO_2_ for 4 h, and subsequently washed three times with 0.01 M PBS per well to remove gentamicin. After the removal of gentamicin and the addition of DMEM, the EBL cells were incubated at 37 °C and 5% CO_2_ for 0, 8, 16, 24, 32, 40 and 48 h. The culture medium was directly added to modified MTA plates at each of the above-mentioned incubation periods to quantify the number of extracellular PG45 bacteria (Figure 2A). The EBL cells were then collected after being digested with 0.25% trypsin with EDTA after 5 min, and they were centrifuged at 10,000× *g* for 10 min. After resuspension with 1 mL modified MTB, the culture medium was directly added to modified MTA plates; this was used to calculate the amount of intracellular PG45. Three replicates were conducted for each group.

### 2.5. MTA Plate Counts Test

In order to investigate whether the invasion of non-phagocytic cells by *M. bovis* has cell or bacterial strain tropism, we selected MDBK, BT, EBL and EBTr cells as four types of bovine non-phagocytic cells, and the PG45, 07801, 08M and PG45-GFP strains, to verify whether exogenous GFP protein interferes with the invasion of *M. bovis*. The MDBK, BT, EBL and EBTr cells were cultured in 12-well cell plates and were infected with PG45, 07801, 08M and PG45-GFP (MOI of 1000) at 37 °C with 5% CO_2_. After 4 h of incubation, the unadhered mycoplasmas were washed off with 0.01 M PBS and treated with 400 µg gentamicin at 37 °C for 4 h. The cells were then digested with 0.25% trypsin–EDTA containing Phenol Red and centrifuged at 10,000× *g* for 10 min, and the supernatant was discarded. Then, 50 µL culture solution was diluted on an MTA plate and cultured at 37 °C with 5% CO_2_ for 4 days. We also excluded plates with fewer than 30 or more than 300 single colonies, and the invasion rate was calculated according to the following formula:

(1) $$Xn=Nn\times \frac{1\;mL}{50\;\mu L}\times dilution\;factor$$

(2) $$\bar{X}=\frac{\left(X_{1}+X_{2}+\ldots +X_{n}\right)}{2.5\times 10^{8}n}\times 100\%$$

For each set of experiments, we established n (n = 3) replicated cell wells. Each cell well culture medium was diluted in a gradient to 10^−5^, and each diluted portion was spread onto one MTA plate. Among all the plates, only those with colony counts between 30 and 300 were selected for statistical counting, denoted as Nn. Based on Formula (1), the number of PG45 invading into the cells, *Xn*, was calculated. Finally, the average invasion rate *X* for each set of experiments was calculated using Formula (2).

### 2.6. Confocal Microscopy

To reduce the effect of antibody incubation on *M. bovis* invasion, it was subjected to an immunofluorescence staining test. MDBK, BT, EBL, and EBTr cells were cultured until 70% confluence, and these cells were infected with PG45-GFP (MOI of 1000) in 35 mm dishes at 37 °C with 5% CO_2_ for 4 h. After infection, the unadhered mycoplasmas were washed off with 0.01 M PBS. The cells were fixed with 4% paraformaldehyde and permeabilized with 0.5% Triton X-100 at 25 °C for 10 min. The membranes and nuclei of these cells were labeled using a cell plasma membrane staining kit with DiI (Beyotime, Beijing, China) and 4′,6-diamidino-2-phenylindole (DAPI) (Sigma-Aldrich, St. Louis, MO, USA), respectively. All cells were visualized with a confocal microscope (Leica TCS/SP8, Wetzlar, Germany).

### 2.7. High-Content Live Cell Imaging and Analysis System

EBL cells were inoculated into a 96-well cell culture plate and grown to 40–50% confluence. The membranes of the EBL cells were labeled using DiI (100 µL/well) under incubation at 37 °C for 30 min without light to stain the cell membrane (the staining agent was diluted with cell maintenance medium according to the instructions, and the cell maintenance medium was DMEM containing 5% FBS). After staining, the supernatant was discarded, and the cells were washed with PBS 3 times. Then, we used Hoechst 33,342 Staining Solution for Live Cells (Beyotime, Beijing, China) to stain the nuclei, incubating it at 37 °C for 10 min without light (the staining solution was diluted with cell maintenance medium according to the instructions). After staining, the supernatant was discarded, and the cells were washed with PBS three times. Finally, 100 µL PG45-GFP (MOI of 1000) was added to each well. The dynamics of living cells and the green fluorescence signals were monitored on the High Content Analysis System (PerkinElmer, Operetta CLS, Waltham, MA, USA), and photos were taken continuously for 6 h.

### 2.8. Transmission Electron Microscopy

The concentration of MDBK, BT, EBL and EBTr cells was adjusted to 5 × 10^7^ cells/mL using a cell enumerator, and the cells were inoculated in a T75 cell culture bottle and cultured in a cell temperature chamber containing 5% CO_2_ at 37 °C for 18–24 h. When the cells grew to 80–90% confluence, they were infected with PG45 (MOI of 1000) at 37 °C with 5% CO_2_ for 4 h. After infection, the adherent cells were scraped off with a cell scraper and centrifuged at 3000× *g* for 5 min, and the supernatant was discarded. Ultrathin sections were prepared by slowly adding 3% glutaraldehyde overnight and were observed under a transmission electron microscope (LTD. HT7700).

We created ultrathin sections of EBL cells at different time periods (30, 60, 90, 120, 180 and 240 min), and they were infected with PG45 in order to observe the invasion process of *M. bovis*.

### 2.9. Endocytosis Inhibitor Test

The EBL, MDBK, EBTr and BT cells were inoculated into 24-well cell plates at 1.0 × 10^5^ cells/pores. When the cell growth reached 70% to 80% confluence, different concentrations of endocytosis inhibitors were added: Chlorpromazine (CPZ) (5 µM, 10 µM, 20 µM), Amiloride (AMI) (20 µM, 40 µM, 60 µM) and Methyl-β-cyclodextrin (M-β-CD) (2 µM, 4 µm, 6 µm). PBS was used as a negative control, and the cells were cultured at 37 °C with 5% CO_2_ for 1 h. Subsequently, PG45 was added at an MOI of 1000. The infection rate of PG45-infected cells was determined via the plate counting method. At the same time, the effects of the endocytosis inhibitors on cell proliferation and toxicity were detected with a Cell Counting Kit-8 (Med Chem Express, Shanghai, China). The above experiment was repeated three times for each working concentration.

### 2.10. siRNA Transfection and Invasion Assays

An siRNA oligo sequence (5′-GGGCCAGCUAAACAAAUAUTT-3′; 5′-AUAUUUGUUUAGCUGGCCCTT-3′) was designed based on bovis clathrin heavy chain (GeneID: NM_174023.2), and a negative control siRNA was synthesized by Sangon Biotech. EBL cells were seeded on 35 mm dishes, transfected with the siRNA and cultured via Opti-MEM (Thermo Fisher Scientific) for 36 h. The knockdown efficiency was quantified via Western blotting. Following treatment with the siRNA, the EBL cells were infected with PG45, 07801 and 08M (MOI of 1000) and subsequently subjected to the antibiotic protection assay, and the invasiveness was determined.

We used confocal laser microscopy to observe the change in the PG45 invasion rate after the knockdown of clathrin (CLR) using the siRNA. After PG45-GFP had infected the EBL cells for 4 h, the cells were incubated with clathrin heavy-chain rabbit mAb (Abclonal, Wuhan, Hubei, China) at a dilution of 1:200, at 37 °C for 1 h, and subsequently incubated with goat anti-rabbit 561 fluorescent secondary antibodies (1:5000) for 2 h. Nuclear staining was carried out using DAPI at 37 °C for 10 min. The samples were observed under a laser confocal microscope.

### 2.11. Statistics Analysis

All statistical analyses were performed using GraphPad Prism version 7.0. Student’s *t*-test or ANOVA was used for multiple comparisons. The intracellular fluorescence intensity was analyzed via ImageJ.1.45 bundled with 64-bit Java (In addition to the displayed areas, we selected all areas of at least 3 photos for the statistical analysis of each set of data). * *p* < 0.05; ** *p* < 0.01; *** *p* < 0.001; NS, no significant difference (*p* > 0.05).

## 3. Results

### 3.1. Screening Results of Optimal Conditions for M. bovis Invasion Cells

**Figure 1:**
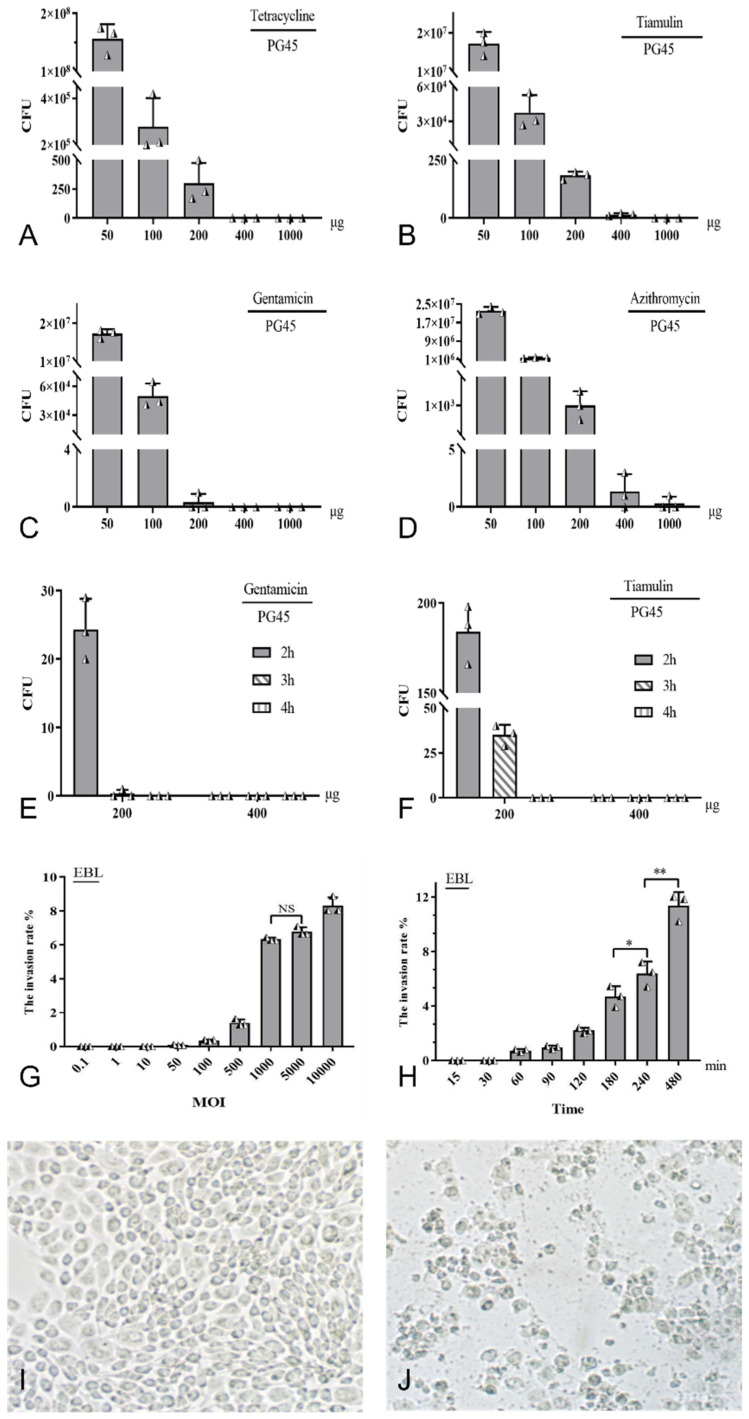
Optimal selection of MBC and MOC. Different concentrations of Tetracycline, tiamulin, gentamicin, and azithromycin were added to the culture medium containing PG45. Tetracycline (**A**) and gentamicin (**C**) with a working concentration of 200 µg/mL to 400 µg/mL, as well as azithromycin (**D**) and tiamulin (**B**) with a working concentration of 400 µg/mL to 1000 µg/mL, can all effectively kill PG45. Under the condition of acting for 3 h, both gentamicin (**E**) and tiamulin (**F**) with a working concentration of 400 µg/mL can completely kill PG45. Different MOIs of PG 45 were added to EBL cells, when it increased to 100 of MOI, the invasion rate of PG45 began to be statistically significant (**G**). PG45 was added to EBL cells at an MOI of 1000, and the invasion rate of PG45 was measured by observing different interaction times (**H**). The cell morphology of PG45-infected EBL cells with an MOI of 1000 for 240 min, the morphology of EBL was essentially normal (100×) (**I**). After 240 min of infection with PG45 at an MOI of 10,000, EBL cells exhibited wrinkling and cytopathic effects (100×) (**J**).* *p* < 0.05; ** *p* < 0.01; NS, no significant difference (*p* > 0.05).

Tetracycline, tiamulin and gentamicin with working concentrations of 200 µg/mL to 400 µg/mL and azithromycin with working concentrations of 400 µg/mL to 1000 µg/mL could effectively kill PG45 (Figure 1A–D). PG45 could be completely killed by gentamicin and tiamulin with a working concentration of 400 µg/mL acting for 3 h, and the effect of gentamicin was better than that of tiamulin (Figure 1E,F).

As the MOI increased, the invasion rate of PG45 in EBL cells was also elevated. When it increased to 100, the invasion rate became statistically significant. When the MOI increased to 5000 from 1000, the increase in the invasion rate was not significant (*p* > 0.05) (Figure 1G). It is noteworthy that, although the invasion rate was the highest when MOI = 10,000 (Figure 1J), compared to MOI = 1000 (Figure 1I), the superinfection caused significant cell differentiation, with some cells shrinking and exhibiting other changes (Figure 1J). These changes were likely to lead to more experimental errors. Accordingly, 1000 was considered to be the optimal MOI.

On the basis of the optimization of the MOI, the infection time was also optimized. The results showed that the invasion rate of *M. bovis* was significantly increased (*p* < 0.05) at 240 min (Figure 1H). Although the invasion rate was significantly higher at 480 min than at 240 min (*p* < 0.01), the experimental period was long. Experimental errors can be caused by mycoplasma reproduction. Therefore, 240 min was chosen as the optimal invasion time in this study. In conclusion, after the optimization of both the MOI and invasion time, combined with the observation results of the cell morphological changes, an MOI of 1000 and an invasion time of 240 min were considered to be the optimal invasion conditions.

### 3.2. M. bovis Survival Inside Cells

**Figure 2:**
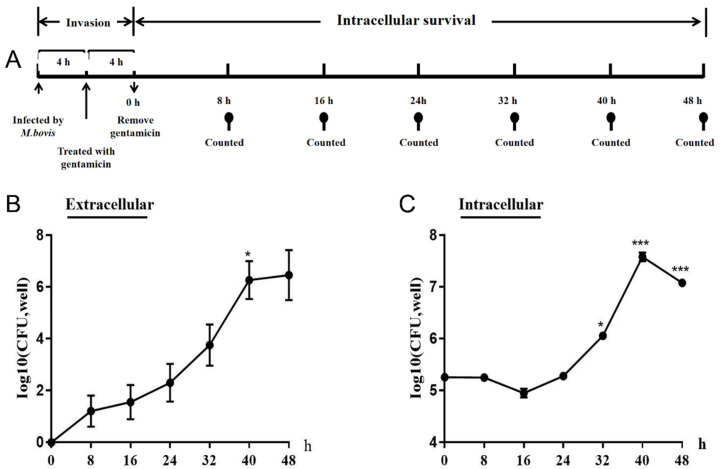
Protocol and results of *M. bovis* intracellular survival test. (**A**) Intracellular viability assay protocol of *M. bovis*. (**B**) CFUs of extracellular PG45 after different culture periods: the number of *M. bovis* bacteria increased gradually with the passage of time, and there was a significant difference at 40 h compared to 32 h (*p* < 0.05). (**C**) CFU of intracellular PG45 after different culture periods: *M. bovis* had a significant difference at 32 h (*p* < 0.05) and an extremely significant difference at 40 h and 48 h compared to 0 h (*p* < 0.01). * *p* < 0.05; *** *p* < 0.001.

The extracellular counting results showed that no *M. bovis* bacteria were detected at 0 h, proving that all extracellular *M. bovis* bacteria were killed after incubation with the antibiotics. Extracellular living mycoplasmas were detected after 8 h, and *M. bovis* reproduced rapidly over time (Figure 2B). The intracellular counting results showed that the total CFU remained essentially unchanged when *M. bovis* invaded the cells within 0–24 h, and it increased rapidly thereafter. The CFU increased significantly (*p* < 0.05) at 32 h and reached a peak at 40 h. The amount of intracellular PG45 decreased at 48 h, but there was still a significant difference (*p* < 0.01). Thereafter, the number of PG45 bacteria decreased significantly (*p* < 0.01) (Figure 2C).

### 3.3. Invasion Rate in Different Cells According to Plate Count Test

**Figure 3:**
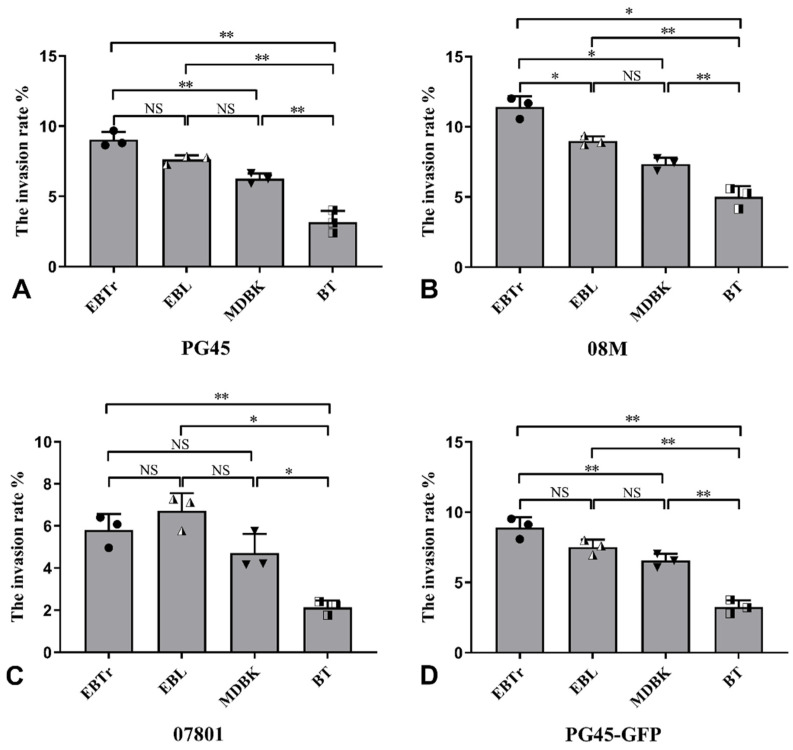
Invasion rates of four mycoplasma strains into various cell types. (**A**) PG45 exhibited the highest invasion rate in EBTr cells and the lowest in BT cells. (**B**) 08M. (**C**) 07801. (**D**) PG45-GFP. * *p* < 0.05; ** *p* < 0.01; NS, no significant difference (*p* > 0.05).

The invasion rates of the four strains of PG45, 08M, 07801 and PG45-GFP were the highest in EBTr cells compared to EBL, MDBK and BT cells. In addition, the invasion rate in BT cells was significantly lower than that in the remaining three cell types (*p* < 0.01). The invasion rate in EBTr cells was significantly higher than that in MDBK cells (*p* < 0.01), but the difference was not significant (*p* > 0.05) compared with EBL cells (Figure 3A–D). It is worth noting that the invasion rates of PG45 and PG 45-GFP remained consistent across all four cell types, suggesting that the introduction of GFP green fluorescent proteins has no obvious effect on the invasion rate of mycoplasma (Figure 3A,D). PG45, 08M and 07801 have the ability to invade a variety of non-phagocytic cells, and the invasion ability of 07801 is slightly weaker than that of PG45 and 08M.

### 3.4. Observation Results of Confocal Microscopy

**Figure 4:**
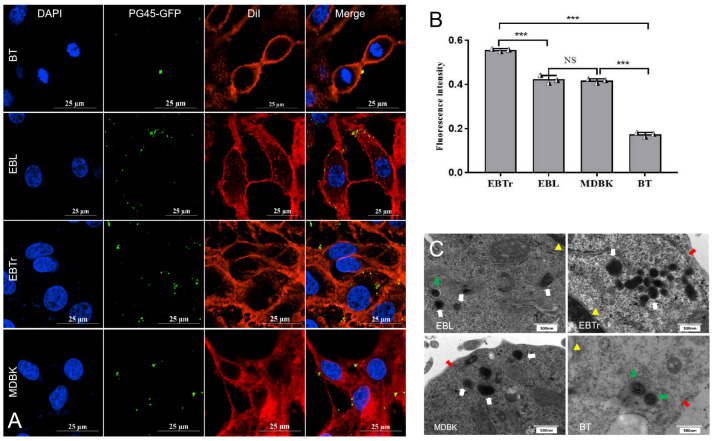
Invasion of four cell types by the PG45-GFP. (**A**) Laser confocal microscopy images of PG45-GFP strain invading MDBK, BT, EBL and EBTr cells. The red fluorescence represents the cytoplasm and cell membrane, the green fluorescence represents mycoplasma PG45-GFP, and the blue fluorescence represents the cell nucleus. (**B**) Quantification of intracellular green fluorescence intensity across the four cell types, revealing that the mean intracellular fluorescence intensity in EBTr cells was significantly higher compared to the other three cell types (*p* < 0.01), while BT cells exhibited significantly lower mean intracellular fluorescence intensity (*p* < 0.01). (**C**) Transmission electron microscopy images show PG45 invading various cells, with the white arrow indicating PG45 in the cytoplasm, the green arrow pointing to PG45 in endosomes, the red bidirectional arrow marking the cell membrane, and the yellow triangle denoting the nuclear membrane. *** *p* < 0.001; NS, no significant difference (*p* > 0.05).

Confocal microscopy revealed that PG45-GFP could invade the four types of non-phagocytic cells (Figure 4A). Further statistical analysis of the intracellular green fluorescence showed that the invasion rate of PG45-GFP varied in different cells. The intracellular fluorescence intensity was in the order of EBTr, EBL, MDBK and BT from high to low. Regarding the mean intracellular fluorescence intensity, that of EBTr cells was significantly higher than that of the remaining three cell types (*p* < 0.01). The mean intracellular fluorescence intensity of BT cells was significantly lower than that of the remaining three cell cells (*p* < 0.01). Meanwhile, the difference in the intracellular fluorescence intensity was not significant (*p* > 0.05) between the EBL and MDBK cells (Figure 4B).

### 3.5. Observations from Transmission Electron Microscopy

After PG45-GFP had infected the MDBK, BT, EBL and EBTr cells (MOI of 1000; infection time of 240 min), TEM observation showed that PG45 was mostly found directly in the cytoplasm (Figure 4C, as indicated by white arrows) and some *M. bovis* bacteria were present in the endosomes (Figure 4C, as indicated by green arrows). The experimental results directly indicated that PG45 invaded a variety of non-phagocytic cells and existed abundantly in the cytoplasm.

### 3.6. Invasion Process of PG45-GFP According to Confocal Microscopy

**Figure 5:**
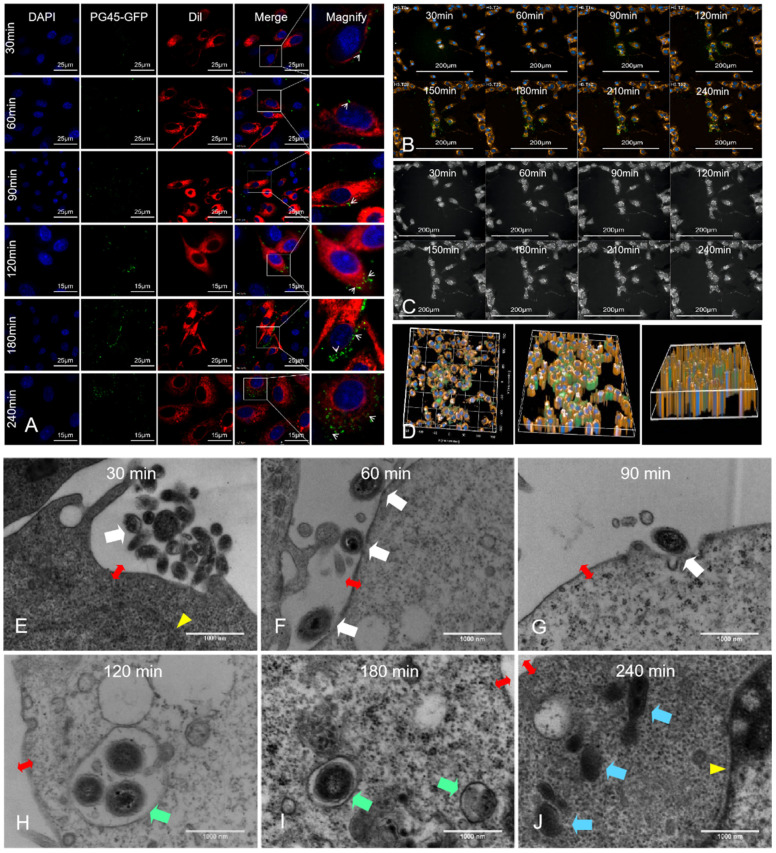
The process of PG45 invading EBL cells. (**A**) Laser confocal microscopy images capturing the invasion of EBL cells by PG45 at various time points. The red fluorescence represents the cytoplasm and cell membrane, the green fluorescence represents mycoplasma PG45-GFP, and the blue fluorescence represents the cell nucleus. (**B**) High-content video screenshots reveal that PG45 started adhering to the cell surface at 60 min, with significant adhesion and minor cytoplasmic entry at 90 min, which progressively increased over time. (**C**) Schematic diagram illustrating the intracellular positions of PG45, color-coded to show the gradual increase in PG45 over time. (**D**) 3D map of PG45 intrusion at 4 h, demonstrating the presence of green fluorescent PG45 within the yellow cytoplasm in all three views. (**E**–**J**) Sequential electron microscopy images showing the interaction between *M. bovis* and EBL cells: (**E**) Large numbers of *M. bovis* surrounding the cell membrane at 30 min; (**F**) PG45 beginning to bind tightly to the cell membrane at 60 min; (**G**) Cell membrane invagination induced by PG45 at 90 min; (**H**) PG45 entering the cell and residing in endosomes at 120 min; (**I**) PG45 located within intracellular vesicles at 180 min; (**J**) PG45 escaping from vesicles and residing in the cytoplasm at 240 min. The white arrow indicates PG45 in the cytoplasm, the green arrow indicates PG45 in the endocytic vesicles, and the blue arrow indicates PG45 escape from the endocytic vesicles, respectively, while the red bidirectional arrow denotes the cell membrane, and the yellow triangle represents the nuclear membrane.

According to the observation results obtained through laser confocal microscopy for PG45-GFP in EBL cells at different time points, it was found that PG45-GFP greatly adhered to the surfaces of EBL cells within 30 min of infection (Figure 5A, as indicated by white arrows). However, it did not significantly invade the cells. At 60 min after infection, a small number of PG45-GFP had crossed the cell membrane barrier and invaded the cells (Figure 5A, as indicated by white arrows). After 90–120 min infection, the number of intracellular mycoplasmas had increased significantly. At 240 min after infection, several PG45-GFP had successfully invaded the cells. These results coincided with the plate counting results.

### 3.7. Invasion Process of PG45-GFP According to High-Content Live Cell Imaging System

A high-content live cell imaging system was used to capture videos of PG45-GFP’s invasion in this study, with continuous shots taken over 4 h, beginning at the 30-min mark. Afterwards, one screenshot was taken every 30 min and used for a paper presentation. The results revealed that *M. bovis* began to adhere to the cell surface, with green fluorescence located on the outer border of the red membrane at 60 min (Figure 5B). At 90 min, the PG45 that adhered to the lateral cells had significantly increased. Thereafter, the green fluorescence signal in the cytoplasm was gradually enhanced as the infection period progressed. The tracing plot showed that most of the *M. bovis* bacteria tended to move towards the nucleus. The cell contour diagram (Figure 5C) showed that the number of mycoplasma-simulated particles was significantly increased in the cytoplasm, suggesting that *M. bovis* invaded the cytoplasm to a significantly greater extent. Three-dimensional views were obtained via multi-slice scanning. As shown in the graph, a large amount of *M. bovis* successfully invaded the cytoplasm 4 h after *M. bovis* had invaded the cells (Figure 5D; Supplementary Video S1).

### 3.8. Invasion Process of PG45 According to TEM

The electron microscopy observation of PG45 invading the EBL cells at different time points showed that a large amount of PG45 accumulated near the outer surface of the cell membrane at 30 min (Figure 5E, as indicated by white arrows). No PG45 was observed to have invaded the cells in the samples in the visual field of the electron microscope, indicating that the majority of the PG45 had not yet successfully passed through the cell membrane barrier (Figure 5E; cell membrane boundaries as indicated by red bidirectional arrows).

At 60 min after infection, most PG45 membranes were tightly bound to the cell membranes (Figure 5F). At 90 min after infection, most PG45 had achieved the invagination of the host cell membranes (Figure 5G). At 120 min after infection, most PG45 had successfully passed through the cell membrane barrier and had completely invaded the cells and were present in the endosomes (Figure 5H). At 180 min after infection, it was clear that most PG45 had invaded the cells and most of them were located in the vesicles (Figure 5I). At 240 min after infection, all PG45 had escaped from the endosomes and were directly present in the cytoplasm (Figure 5J). At this point, the intracellular mitochondrial lesions were obvious. Some mitochondrial ridges had ruptured, and more autophagosomes appeared inside the cell, suggesting that PG45 may lead to cytotoxicity after invading cells.

### 3.9. Effect Test of Different Endocytic Inhibitors

**Figure 6:**
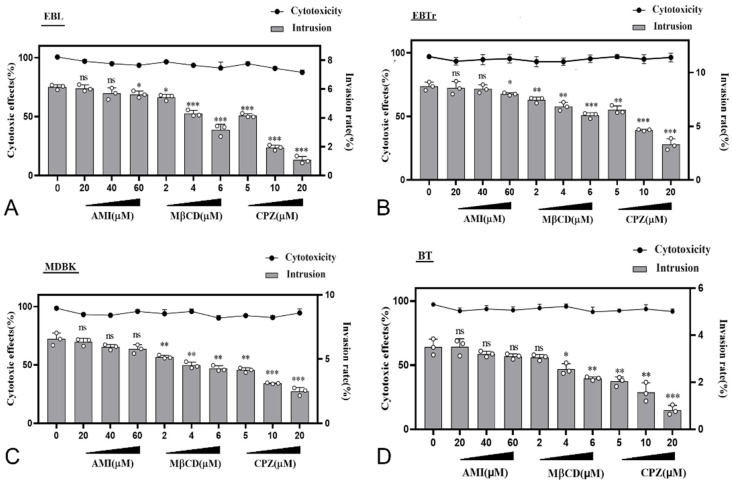
Detection of invasion rate, cell proliferation, and cytotoxicity following treatment with various agents. The horizontal axis represents the concentrations of different inhibitors, the left vertical axis represents cytotoxic effects, and the right vertical axis represents the invasion rate. Cell proliferation activity is negatively correlated with cytotoxicity. (**A**) The results obtained for EBL cells after treatment with different inhibitors, assessing invasion rate, cell proliferation, and cytotoxicity; (**B**) Similar assessments for EBTr cells; (**C**) MDBK cells; and (**D**) BT cells. The findings indicate a significant reduction in the invasion rates of all four cell types when treated with the inhibitor Chlorpromazine (CPZ) (*p* < 0.01). Additionally, a significant decrease in the invasion rates of these cell types was observed when treated with the inhibitor Methyl-β-cyclodextrin (M-β-CD) at concentrations exceeding 4 µM (*p* < 0.01). * *p* < 0.05; ** *p* < 0.01; *** *p* < 0.001; ns, no significant difference (*p* > 0.05).

The experimental results regarding the inhibitory effects of different inhibitors revealed that the invasion rate of PG45 in EBL, EDBK, EBTr and BT cells was significantly reduced (*p* < 0.01) compared with each negative control after inhibiting the clathrin endocytic (CDE) pathway via CPZ. When the working concentration of CPZ was 20 µM, the difference in the invasion rate in the cells was extremely significant (*p* < 0.001). When the working concentration of M-β-CD was higher than 4 µM, the difference in the invasion rate reduction was significant (*p* < 0.01). After inhibiting different macropinocytosis pathways with AMI, it generally failed to lead to a significant decrease in the PG45 invasion rate (Figure 6A–D). The results of the inhibitors regarding cell proliferation and toxicity showed that none of the three inhibitors significantly affected cell proliferation or caused significant cytotoxicity at each working concentration (Figure 6A–D).

### 3.10. Experimental Results of Clathrin siRNA Interference

**Figure 7:**
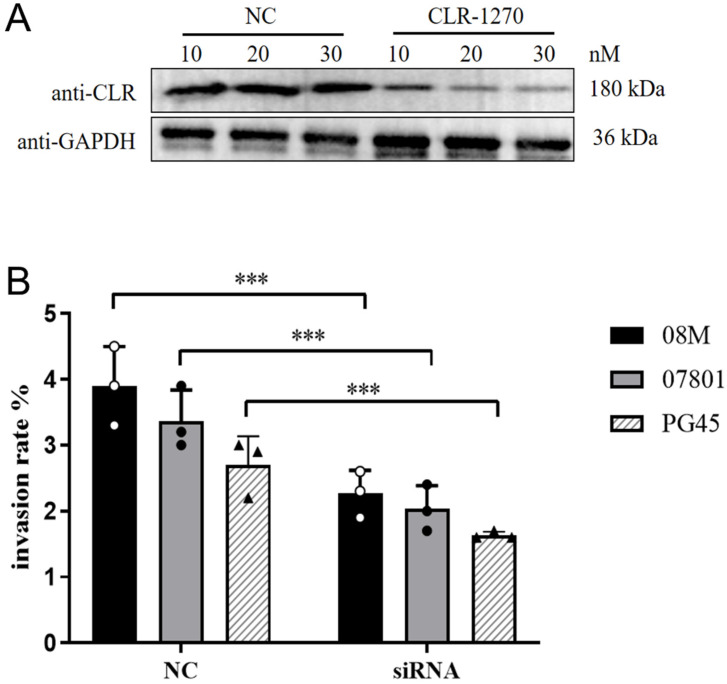
The results of intrusion rate detection for *M. bovis* following siRNA knockdown of CLR. (**A**) Assessment of the interference effects of CLR-1270 siRNA oligo on clathrin (CLR) in EBL cells, revealing that the optimal interference effect is achieved at a working concentration of 20 nM. (**B**) Following the siRNA knockdown of CLR, a significant decrease (*p* < 0.001) was observed in the intrusion rates of *M. bovis* strains, specifically PG45, 07801, and 08M. *** *p* < 0.001.

After treating EBL cells with CLR-1270 oligo (working concentration of 20 nM (Figure 7A)), the invasion rates of the three mycoplasmas, PG45, 07801 and 08M, were significantly decreased (*p* < 0.001). However, their invasion was not completely blocked (Figure 7B). The findings suggested that the CLR of host cells played an important role in *M. bovis* invasion. Meanwhile, there are other CLR-independent invasion routes.

## 4. Discussion

### 4.1. Ability of M. bovis to Invade Host Cells

Chronic infection is not only an important feature of *M. bovis* but also one of the main reasons why it is difficult to end its epidemic. In recent years, with the in-depth study of the pathogenic mechanism of *M. bovis*, scientists have generally concluded that *M. bovis* invading cells through immune evasion is one of the main causes of chronic infection [15,16]. For a long time, it was widely believed that mycoplasma was strictly a prokaryotic organism and did not have the ability to invade host cells. However, with the development of modern biotechnology, numerous findings have indicated that mycoplasmas can invade or internalize a variety of non-phagocytic cells [7,17,29]. It has been proven that mycoplasma penetrans was one of the earliest mycoplasmas capable of invading host cells. Moreover, with continuous in-depth research, scientists have discovered that some other intact mycoplasmas are colonized in the respiratory tract and/or urogenital tract, such as *M. fermentans*, *M. pneumonia* and *M. bovis*, which can simultaneously enter host cells and exist throughout the cytoplasm and perinuclear region [30,31,32,33].

In this assay, PG45 was regarded as the *M. bovis*-type strain and EBL cells as the model cells to determine the minimum bactericidal concentration (MBC) and multiplicity of infection (MOI), because PG45 is the most commonly used standard strain for the study of *M. bovis*, and its whole-genome sequence and related background are relatively clear. EBL cells are also the most commonly used cell line to study mycoplasma and are easier to culture and preserve [29,34]. The four cell lines selected for this experiment are all derived from epithelial cell lines. MDBK is isolated from bovine kidney tissue, EBL originates from bovine embryonic lung tissue, BET cells are derived from bovine embryonic tracheal tissue, and BT cells are isolated from bovine turbinate tissue. Since *Mycoplasma bovis* has a greater tendency to invade the respiratory system, we conducted experiments using the aforementioned three cell lines related to the respiratory system, while using MDBK cells as a control to observe whether *Mycoplasma bovis* has a preference for invading these cell types. It is worth noting that the invasion rates of the three *M. bovis* strains in the four types of bovine-derived non-phagocytes were different, including those in MDBK and EBTr cells. Our analysis suggests that a possible reason for this difference is the tissue tropism of the strains because PG45 was isolated from bovine mastitis samples, while both 07801 and 08M were isolated from bovine pneumonia samples. Secondly, it may be related to the differences in the ECM components on the surfaces of different cell membranes. However, the specific reasons still need to be further analyzed. It should be pointed out that EBTr cells were difficult to culture in this study. Therefore, both EBL and MDBK cells should be used as the main model cells in subsequent related research.

### 4.2. Pathways by Which M. bovis Invades Host Cells

This bacterium employs various strategies to evade host immune responses, including cell invasion. Understanding the mechanisms of cell invasion is essential in deciphering the pathophysiology of infectious diseases and could lead to the development of novel therapeutic approaches. Nevertheless, the specific mechanism by which *M. bovis* infiltrates bovine cells remains unclear.

Lipid rafts are specialized microdomains within cell membranes, characterized by their enriched content of cholesterol, sphingolipids and specific proteins. These regions are dynamic and play crucial roles in various cellular processes, such as signal transduction, membrane trafficking and cell adhesion [35,36]. Pathogens, both viruses and bacteria, exploit lipid rafts for initial attachment to host cell membranes. The unique lipid composition of rafts facilitates the binding of pathogen surface proteins or glycoproteins to specific receptors on the host cell surface. Once attached, pathogens often induce rearrangements in lipid rafts to trigger endocytic processes [37,38]. This can involve the formation of membrane invaginations or the recruitment of specific signaling molecules that promote the internalization of the pathogen into host cells [39].

Although several articles have reported the phenomenon of *M. bovis* invading non-phagocytes, the invasion pathway of *M. bovis* is less clear, and there are no universally accepted research results so far. CPZ, AMI and M-β-CD were used to inhibit the CDE pathway, macropinocytosis pathway and lipid raft pathway, respectively, in this study. The results show that *M. bovis* can invade various non-phagocytic cells through the CDE pathway and lipid raft pathway, with the CLR-mediated endocytic pathway as its main invasion method. These results are consistent with the findings reported by Nishi et al., indicating that *M. bovis* invades joint synovial cells through the CDE pathway [40]. Unfortunately, Nishi did not investigate the lipid raft pathway. Some scholars have also pointed out that *M. bovis* is too large (200–400 nm) to invade host cells through the CDE pathway. Additionally, there is no key cellular receptor (which is necessary for the CDE pathway) identified so far during *M. bovis* invasion. Therefore, we are unable to completely eliminate the uncertainty in this regard [41].

The transmission electron microscopy results showed that *M. bovis* was abundantly present in the host cell’s vesicle structure at 120 min after invading the cells, but it gradually escaped from the vesicles after 180 min and directly existed in the cytoplasm. Based on the above findings, we are confident that *M. bovis* invades cells through a certain endocytic pathway and may be capable of escaping from the vesicle. However, its mechanism of action and key virulence proteins are still unknown. Therefore, further research and analysis are required.

This study found that *M. bovis* is able to multiply intracellularly by measuring the intracellular and extracellular *M. bovis* counts at different time points after its invasion. Interestingly, *M. bovis* was also detectable extracellularly at 8 h after invasion. The results revealed that *M. bovis* may not only multiply inside the cells but also escape from the intracellular to the extracellular environment through certain pathways after invading EBL cells. The mycoplasma count increases significantly over time. We believe that *M. bovis* can be released from the cell after invasion through certain pathways, or the intracellular mycoplasma can be released by inducing the apoptosis or necrosis of these invaded cells growing in the culture medium. Although mycoplasma contains conservative hemolysin-related genes and has a certain hemolytic ability, there are no literature reports on whether *M. bovis* can utilize its hemolysin genes to achieve its function of lysing cells. This still needs further research [42,43].

### 4.3. Toxic Effect of M. bovis on Cells

Do *M. bovis*-infected cells induce apoptosis? Does it promote inflammation or is it immunosuppressive? These questions have always been controversial in the scientific community. As reported by Gerlic, mycoplasmas may inhibit tumor necrosis factor-α-induced apoptosis in the monocyte U937 cell line [44]. It has been shown that mycoplasma septicemia may induce exosomal gga-miR-193a, which interferes with cell proliferation, apoptosis and cytokine production by targeting the KRAS/ERK signaling pathway [45]. Fang reports that *M. bovis* may delay the increased expression of apoptotic genes in macrophages [46]. At the same time, many literature reports show that various types of mycoplasmas may induce apoptosis and produce cytotoxic effects. For example, Niu’s study suggests that *M*. *putrefaciens* may induce inflammation and apoptosis [47]. Blocking IL-6R attenuates cell apoptosis and regulates the inflammatory response in *M. pneumoniae*-infected A549 cells [48]. This study demonstrates that PG45 leads to significant cell necrosis and apoptosis after it infects the cell. These findings further support the induction of cell death and apoptosis.

Due to the absence of a bovine phagocytic cell line, this study was unable to further explore whether *M. bovis* has the capability to escape from phagocytic endocytic vesicles. This limitation has somewhat impeded our comprehensive understanding of the mechanisms involved in the invasion of cells by *M. bovis* and its survival within them. Additionally, although we endeavored to use siRNA technology to reduce clathrin expression in MDBK, EBTr, and BT cells, with the aim of further elucidating CLR’s role in the invasion process of *M. bovis*, the related experiments did not produce valid data owing to the low transfection rates of these cells.

## 5. Conclusions

The invasion phenomena of three *M. bovis* strains, namely PG45, 07801 and 08M, in four types of cells, namely EBL, MDBK, EBTr and BT cells, were investigated in this study. The results demonstrate that all three *M. bovis* strains can invade these four types of non-phagocytes. Moreover, the different strains vary in their invasion abilities. The results of transmission electron microscopy at different time points of invasion show that *M. bovis* invades the host cell cytoplasm in the form of vesicles at 120 min but escapes from the vesicles by 240 min. *M. bovis* likely employs the CDE pathway or lipid raft pathway to invade host cells, confirming its ability to utilize the CDE pathway for cell invasion.

In our subsequent studies, we intend to concentrate on identifying virulence factors that are closely linked to *M. bovis* invasion of non-phagocytic cells. Furthermore, we aim to delve deeper into the interactions between these virulence factors and host cell proteins, with the goal of uncovering the intricate mechanisms underlying the invasion of cells by *M. bovis*.

## Data Availability

The original contributions presented in this study are included in the article. Further inquiries can be directed to the corresponding author.

## Supplementary Materials

The following supporting information can be downloaded at: https://www.mdpi.com/article/10.3390/pathogens13111003/s1, Video S1: The process of *M. bovis* invasion EBL cells.
